# Genomic epidemiology of Lineage 4 *Mycobacterium tuberculosis* subpopulations in New York city and New Jersey, 1999–2009

**DOI:** 10.1186/s12864-016-3298-6

**Published:** 2016-11-21

**Authors:** Tyler S. Brown, Apurva Narechania, John R. Walker, Paul J. Planet, Pablo J. Bifani, Sergios-Orestis Kolokotronis, Barry N. Kreiswirth, Barun Mathema

**Affiliations:** 1 Department of Medicine, Columbia University, New York, NY USA; 2 Sackler Institute for Comparative Genomics, American Museum of Natural History, New York, NY USA; 3 The Genomic Institute of the Novartis Research Foundation, San Diego, CA USA; 4 Department of Pediatrics, Division of Infectious Diseases, The Children’s Hospital of Philadelphia, Philadelphia, PA USA; 5 Novartis Institute for Tropical Diseases, Singapore, Singapore; 6 Department of Epidemiology and Biostatistics, School of Public Health, SUNY Downstate Medical Center, Brooklyn, NY USA; 7 Tuberculosis Center, Public Health Research Institute, Newark, NJ USA; 8 Department of Epidemiology, Mailman School of Public Health, Columbia University, New York, USA

**Keywords:** *Mycobacterium tuberculosis*, Whole genome sequencing, Phylogenomics, Surveillance

## Abstract

**Background:**

Whole genome sequencing (WGS) has rapidly become an important research tool in tuberculosis epidemiology and is likely to replace many existing methods in public health microbiology in the near future. WGS-based methods may be particularly useful in areas with less diverse *Mycobacterium tuberculosis* populations, such as New York City, where conventional genotyping is often uninformative and field epidemiology often difficult. This study applies four candidate strategies for WGS-based identification of emerging *M. tuberculosis* subpopulations, employing both phylogenomic and population genetics methods.

**Results:**

*M. tuberculosis* subpopulations in New York City and New Jersey can be distinguished via phylogenomic reconstruction, evidence of demographic expansion and subpopulation-specific signatures of selection, and by determination of subgroup-defining nucleotide substitutions. These methods identified known historical outbreak clusters and previously unidentified subpopulations within relatively monomorphic *M. tuberculosis* endemic clone groups. Neutrality statistics based on the site frequency spectrum were less useful for identifying *M. tuberculosis* subpopulations, likely due to the low levels of informative genetic variation in recently diverged isolate groups. In addition, we observed that isolates from New York City endemic clone groups have acquired multiple non-synonymous SNPs in virulence- and growth-associated pathways, and relatively few mutations in drug resistance-associated genes, suggesting that overall pathoadaptive fitness, rather than the acquisition of drug resistance mutations, has played a central role in the evolutionary history and epidemiology of *M. tuberculosis* subpopulations in New York City.

**Conclusions:**

Our results demonstrate that some but not all WGS-based methods are useful for detection of emerging *M. tuberculosis* clone groups, and support the use of phylogenomic reconstruction in routine tuberculosis laboratory surveillance, particularly in areas with relatively less diverse *M. tuberculosis* populations. Our study also supports the use of wider-reaching phylogenomic and population genomic methods in tuberculosis public health practice, which can support tuberculosis control activities by identifying genetic polymorphisms contributing to epidemiological success in local *M. tuberculosis* populations and possibly explain why certain isolate groups are apparently more successful in specific host populations.

**Electronic supplementary material:**

The online version of this article (doi:10.1186/s12864-016-3298-6) contains supplementary material, which is available to authorized users.

## Background

Tuberculosis (TB) epidemiology in New York City has undergone dramatic changes since the resurgent TB epidemic of the 1990s, when over 3000 cases were reported each year between 1991 and 1994, many in outbreak clusters among vulnerable populations [1]. TB incidence is now at an all-time low (7.2 cases per 100,000 people in 2014) [2], outbreak clusters have become increasingly rare, and so-called *endemic clones* have become a major source of new TB infections in the US-born population [3, 4].

Genotyping of *Mycobacterium tuberculosis* (*M. tuberculosis*) clinical isolates is a cornerstone of TB control in New York City. However, conventional genotyping methods (including restriction length fragment polymorphism typing, spoligotyping, and mycobacterial interspersed repetitive Units (MIRU) typing), interrogate less than 0.01% of the approximately 4 Mb *M. tuberculosis* genome and thus lack the discriminatory power to detect small-scale genetic differences within closely related populations. In these situations, genotyping will often yield little if any useful information, even in isolates with wide geographic distribution and long epidemiological histories in a given population [3].

Whole genome sequencing (WGS) directly overcomes these limitations and has rapidly become an important, if not central, research tool in TB epidemiology: WGS-based studies have detected previously unknown outbreak clusters among isolates with identical MIRU-VNTR types [5, 6] and identified so-called super-spreaders responsible for multiple secondary infections in the community [7]. In addition, an expanding body of work has employed WGS data to address a wide-reaching set of previously uninvestigated questions in *M. tuberculosis* evolution and population genomics [8–11].

Next-generation WGS technologies have markedly decreased per-isolate sequencing costs, and are expected to replace many current modalities in public health microbiology [12, 13]. Specific applications of interest for TB control include rapid drug resistance typing, locating cryptic outbreak clusters and transmission hotspots not identified via field epidemiology, and identification and tracking of novel *M. tuberculosis* strains in the community. SNP-distance based strategies have proven useful for identifying recent TB transmission [5] and WGS data has allowed for unprecedented phylogenetic resolution between and within *M. tuberculosis* subpopulations. Population genomics studies in both *M. tuberculosis* and other pathogens have established important linkages between the evolutionary and epidemiological histories of endemic and/or emerging pathogen subpopulations [14, 15]. Specifically, emerging *M. tuberculosis* subpopulations are expected to exhibit low sequence diversity, an excess number of high frequency derived alleles, and potentially harbor strain-specific patterns of positive or purifying genomic selection.

Multiple *M. tuberculosis* strains have emerged from New York City and neighboring New Jersey (NYC-NJ) over the last two decades. For example, *M. tuberculosis* isolates from the S75 group, a low-IS*6110* copy number strain first identified in New Jersey, USA [16] in 2002, circulate within the NYC-NJ area, predominantly among HIV-positive and homeless populations [17]. The drug-susceptible C strain was first reported in NYC, where it has caused outbreaks among at-risk populations and sporadic cases in the general population, and then spread widely across the United States [3]. Both C and S75 strains belong to *M. tuberculosis* Lineage 4, the most widely distributed and successful of the six *M. tuberculosis* global phylogeographic lineages [18] and the most prevalent lineage in the New York City area.

This study uses WGS data from TB isolates collected in New York City and New Jersey between 1999 and 2009, applying both phylogenomic and population genomics methods to identify epidemiologically-relevant subpopulations within this relatively monomorphic local population. These methods identify previously known subpopulations (including S75) retrospectively, suggest useful measures for prospective and real-time identification of newly emerging isolate groups, and yield additional information on adaptation and epidemiological success in *M. tuberculosis* isolates endemic to New York City.

## Methods

### Mycobacterium tuberculosis *isolates*

Seventy one total *M. tuberculosis* full genome sequences were included in this study. Fourty-seven isolates from Lineage 4 were included: 32 isolates from TB cases occurring in New York City and New Jersey between 1997 and 2009, including 9 S75 isolates; 9 additional clinical isolates from Sub-Saharan Africa [19, 20] and North America [21]; and 6 well-characterized laboratory strains [20, 22–24]. Two additional isolates from New York City, from Lineage 1 and Lineage 3, were also sequenced for this study. Sequence data for 19 additional non-L4 isolates, plus 3 isolates from the *M. africanum*-like Lineage 6 and the outgroup *M. bovis*, were obtained from publicly available sources (Additional file 1: Table S1).

### Sequencing, alignment, and SNP calling

WGS data were obtained for 34 previously-unsequenced *M. tuberculosis* clinical isolates (Table 1). Isolates were cultured on Löwenstein-Jensen slants and grown at 37 °C for 3–5 weeks. Sequencing libraries were prepared using TruSeq DNA or Nextera DNA preparation kits (Illumina, San Diego, CA). Raw sequencing reads were generated on the Illumina HiSeq 1000 platform and aligned to the H37Rv reference genome (NC_000962.2) using the Burrows-Wheeler Aligner [25]. Genome assemblies for all isolates were deposited in the NCBI Genbank database (accession numbers are listed in Table 1). All isolates had reads covering >99% of the reference genome, and the lowest mean coverage depth for any isolate was 27×. SNPs were called using a PHRED-scaled quality threshold of 40 (Samtools v0.1.19 [26]) and annotated using snpEff v4 [27]. We excluded from analysis all variants occuring within PE and PPE genes, a family of highly repetitive, GC-rich *M.tuberculosis* genes in which recombination has been observed [28].

**Table 1:** Characteristics of the isolates sequenced in this study

Isolate	Lineage	Location	Year	Reads	Mean read depth	%Genome coverage	Filtered SNPs	Genbank Accession
BE_116771	1	NJ	1999	2,883,388	31.633	0.994	2065	LKMF01000000
BE3_11657	3	NJ	1999	2,934,017	63.728	0.996	1046	LKDN01000000
001_13432	4	NYC	2000	3,027,826	75.362	0.996	1060	LKDO01000000
AH_14271	4	NYC	2001	4,045,981	95.556	0.998	1074	LKDP01000000
AH26_26663	4	NYC	2010	1,356,722	33.169	0.997	1044	LJIQ01000000
AH26_28866	4	S. Africa	2011	1,958,645	23.009	0.994	968	LKMH01000000
AU_8623	4	NYC	1998	6,294,738	71.734	0.996	983	LKMG01000000
BE_10225	4	NJ	1999	1,896,522	47.939	0.994	1049	LJIK01000000
BE_13443	4	NYC	2001	3,669,247	91.597	0.995	1071	LKDQ01000000
BE_14248	4	NYC	2001	2,921,250	70.22	0.995	1077	LKDR01000000
BE_7556	4	NJ	1997	3,580,176	41.681	0.995	961	LJIL01000000
C_913	4	NYC	1992	14,910,476	387.981	0.995	1101	LKMI01000000
C_10367	4	NYC	1999	2,082,141	51.722	0.995	1057	LJIP01000000
C_14229	4	NYC	2001	5,108,155	127.485	0.996	1128	LKDS01000000
C130	4	NYC	1991	2,704,576	64.086	0.996	1057	LJIN01000000
C24_20545	4	NYC	2005	2,856,851	71.852	0.997	1125	LJIM01000000
C28_9319	4	NJ	1998	2,179,814	53.922	0.995	1058	LJIO01000000
C28_9904	4	NJ	1999	1,632,080	39.971	0.994	1019	LJIR01000000
C30_19588	4	NYC	2004	4,631,768	115.895	0.996	1083	LKDT01000000
C34_13853	4	NYC	2001	2,008,488	48.272	0.995	1048	LKHH01000000
C4_16679	4	NYC	2002	3,966,004	96.262	0.996	1075	LKIF01000000
C49_20090	4	NYC	2005	1,966,774	47.421	0.995	1024	LKIG01000000
C53_20899	4	NYC	2006	3,105,243	74.778	0.994	1062	LKIH01000000
H_13559	4	NYC	2001	3,185,904	76.306	0.996	1041	LKII01000000
H_13571	4	NYC	2001	1,815,449	44.429	0.995	1021	LKIJ01000000
H_7300	4	NYC	1997	2,011,143	48.599	0.994	1020	LKDL01000000
H55_24991	4	NYC	2009	1,743,190	42.094	0.995	1041	LKIK01000000
H6_10443	4	NJ	1999	1,799,336	43.72	0.995	1019	LKIL01000000
H6_12226	4	NJ	2000	4,457,191	105.789	0.996	1074	LKIM01000000
H6_7420	4	NJ	1997	1,719,494	43.153	0.994	1041	LKDM01000000
I_15762	4	NYC	2002	2,785,341	66.101	0.995	1057	LKIN01000000
KI_19771	4	NYC	2004	2,884,815	69.225	0.995	1079	LKIO01000000
L_13621	4	NYC	2001	8,967,725	221.783	0.997	1098	LKIP01000000
V_13678	4	NYC	2001	1,517,250	35.643	0.997	1018	LKIQ01000000

### Availability of data and materials

The dataset supporting the conclusions of this article is available in the NCBI Genbank repository (http://www.ncbi.nlm.nih.gov, BioProject: PRJNA288586) and supporting sequence alignments and phylogenetic tree data are available on TreeBASE.

### Phylogenetic reconstruction

Phylogenetic trees were estimated using maximum likelihood methods in the POSIX-threads build of RAxML v8 [29]. Node robustness was assessed with 1000 bootstrap pseudoreplicates and a consensus network was calculated [30] as implemented in SplitsTree v4.3.1 [31]. A custom Perl script was used to identify SNPs with alleles unique to a given lineage or subpopulation.

### Neutrality statistics and selection analysis

Neutrality statistics (including Tajima’s *D*, Fu and Li’s *D* and *F*, Ramos-Onsins and Rozas’s *R*_*2*_, and Fay and Wu’s *H*) were calculated in DnaSP v5.10.1 [32] with statistical significance assessed with 10,000–50,000 coalescent simulations. Fay and Wu’s *H* is particularly useful for distinguishing whether a given departure from neutrality is attributable to recent population expansion or a selective sweep [33]. The gene-wise ratio of the nonsynonymous substitution rate to the synonymous substitution rate (dN/dS) was estimated for every gene in the *M. tuberculosis* genome across all phylogenetic branches using the branch-site random effects likelihood (BSREL) model as implemented in HyPhy v2 [34, 35]. This model tests for branch-specific instances of episodic diversifying selection on every internal and terminal branch on the phylogenetic tree (in this case for every single gene fitted on the phylogenomic tree) and, following a likelihood-ratio test and Holm’s correction for multiple tests, detects branches on which a proportion of the codons have evolved under a dN/dS ratio that is significantly different from that of the rest of the codons. The advantage of this model over other so-called branch-site models is that it does not constrain the tree on either sides of the branch being tested to be subject to diversifying selection (foreground branches) and purifying selection (background branches).

## Results

### Population structure and genetic diversity

Maximum likelihood phylogenomic reconstruction based on 14,601 quality-filtered SNPs recovered primary phylogeographic Lineages 1–6 and identified at least six distinct subpopulations within L4 isolates, including S75. Nucleotide diversity (π, the mean number of pairwise nucleotide differences per site [36]) ranged from 1.5E-5 to 1.7E-4 (Table 2), consistent with prior estimates of genetic diversity within coding regions of the *M. tuberculosis* genome [19]. S75 strains were separated from any other L4 isolate by at least 143 SNPs and exhibited lower nucleotide diversity and lower mean pairwise SNP distances between isolates (66.4 vs. 392.8 SNPs for NYC isolates not in the S75 cluster).

**Table 2:** Genetic diversity and neutrality test statistics by lineage (L1-L4)

Lineage	N	S	π	*D*_*T*_	*D*_*FL*_	*F*_*FL*_	*R*_*2*_	*H*_*FW*_	*Hn*_*FW*_
L1	7	2238	1.7E-4	−1.157	−0.9802 (−1.7817*)	−1.0910 (−1.9267*)	0.0812**	457.62	1.180
L2	9	2240	1.3E-4	−1.627	−1.66780 (−2.3468**)	−1.8172 (−2.5742**)	0.0795**	162.92	0.430
L1/L2/L3	19	6249	2.7E-4	−1.622	−2.1420* (−2.9363**)	−2.2253* (−2.9868**)	0.0631**	635.80	0.671
L4	47	4892	1.1E-4	−2.205*	−4.3046** (−5.3287**)	−4.1559** (−4.8682**)	0.0334**	304.47	0.490
L4: NYC	21	2262	8.9E-5	−1.649	−2.5102* (−3.3684 *)	−2.6277* (−3.4054 *)	0.0593**	230.01	0.7874
L4: S75	9	149	1.5E-5	−0.498	0.07218 (0.45497)	−0.07462 (0.22079)	0.1297	−29.14	−1.125

N, number of ingroup sequences; S, number of segregating sites; π, nucleotide diversity; *k*, average number of nucleotide differences; *D*_*T*_, Tajima’s D; *R*_*2*_ *,* Ramos-Onsins and Rozas’ *R*_*2*_, *D*_*FL*_ and *F*_*FL*_, Fu and Li’s D and F (calculated with *M. bovis* as an outgroup); *H*_*FW*_, Fay and Wu’s *H*; *Hn*_*FW*_, Fay and Wu’s normalized *H*. Statistical significance was assess with 10,000 coalescent simulations (50,000 simulations for *R*_*2*_). *P < 0.05, **P < 0.005

### Drug resistance-associated polymorphisms

Polymorphisms at drug resistance-associated codon sites were evaluated for 36 known drug resistance genes (Additional file 2: Figure S1). Mutations in *katG*, which confer resistance to isoniazid, were common among isolates from Lineages 1–3, 5, and 6 and L4 isolates from western and sub-Saharan Africa, and rare among L4 isolates from N. America and Europe, occurring in only a single isolate from this group. S75 isolates were found to have a strain-specific mutation in *embA* (Ala462Val) previously associated with ethambutol resistance [37], however the S75 isolates included in this study are ethambutol-sensitive. L4 isolates from Kwazulu-Natal, South Africa carried drug resistance-associated mutations in *katG*, *rpoB*, *pncA*, and *rrs*, consistent with prior studies on these drug-resistant isolates [38].

### Subgroup-defining polymorphisms

**Fig. 1:**
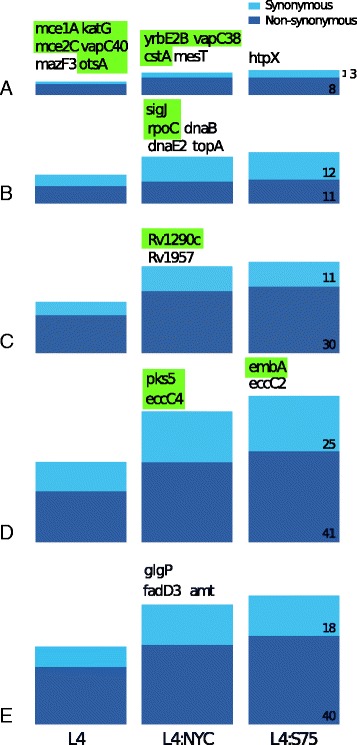
Synapomorphic polymorphisms by functional category and isolate subgroup. **a** Virulence and adaptation, **b** Regulatory and information pathways, **c** Conserved proteins without known function, **d** Cell wall and lipid metabolism, **e** Intermediary metabolism and respiration. L4 includes all (*n* = 47) Lineage 4 isolates included in this study, NYC-NJ (*N* = 32) includes L4 isolates collected in New York City or New Jersey, USA, including the S75 outbreak cluster, and S75 (*N* = 9) includes isolates belonging the New Jersey outbreak cluster described in the text. Genes carrying diagnostic SNPs with known functions in virulence, growth, and/or adaptation are listed above each column, and of these genes, those with non-synonymous polymorphisms are highlighted in yellow. The number of total diagnostic SNPs unique to S75 (which includes those unique to L4 and NYC-NJ) are listed in the third column

**Fig. 2:**
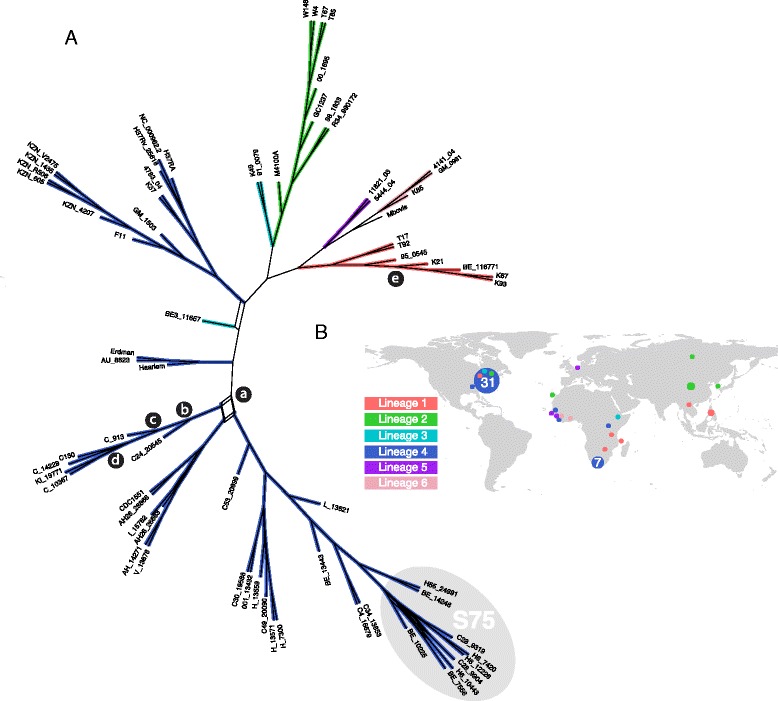
**a** Consensus network of 1000 maximum likelihood bootstrap replicates for *Mycobacterium tuberculosis* isolates from North America, Sub-Saharan Africa, and Asia (*n* = 71) based on 14,601 SNPs. Branches are color-coded by lineage. Isolates from the S75 cluster, identified in New Jersey in 1997–2001, are highlighted; **b** World map of isolate collection locations color-coded by lineage

One hundred seventeen synapomorphic SNPs (i.e. loci at which isolates in given subgroup carry one allele and all isolates outside the subgroup carry a different allele) differentiate L4 isolates from the non-L4 isolates included in this study (Fig. 1). Seventy-five additional SNPs differentiate North American isolates (isolates distal to Node *a* in Fig. 2, including those from New York, New Jersey, and the CDC1551 outbreak strain) from non-North American isolates, and 16 SNPs differentiate S75 from other North American isolates. Synapomorphic SNPs are unequally distributed by functional category, predominantly occurring in genes associated with cell wall functions, lipid metabolism, respiration, and intermediary metabolism. Non-synonymous synapomorphic SNPs occur in multiple genes with known or proposed functions in virulence, growth, and/or adaptation, including known virulence factors (*mce1A*, *mce2C*, *vapC40*, *vapC38*, *otsA*, *yrbE2B* *,* and cstA [39–43]), and also components of gene-regulatory (sigJ, ramB), lipid metabolism (pks5, *fadD15*, Rv3087), intermediary metabolism (*lpdA*), and cell-wall associated pathways (*eccC4*) with known or proposed functions in *M. tuberculosis* virulence [44–50]. New York City and S75 isolates carry a unique non-synonymous mutation in Rv1290c, a conserved gene of unknown function that when disrupted causes a severe attenuation of virulence [51]. Additional file 3: Table S2 lists the complete set of subgroup-defining SNPs.

### Neutrality test statistics and population size expansion

**Fig. 3:**
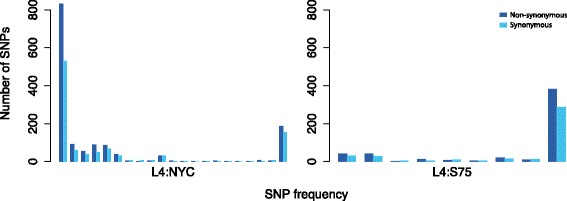
Unfolded site frequency spectra for isolates from the S75 outbreak cluster (L4:S75) and non-S75 isolates from the New York City area. Dark and light blue bars indicate the number of non-synonymous and synonymous SNPs at each SNP allele frequency (from singletons on the *left* to SNPs at fixation on the *right*)

Site frequency-based neutrality test statistics were calculated using whole-genome polymorphism data by lineage (L1-L4) and by subgroups within L4, including the S75 outbreak cluster and non-S75 isolates from New York City and New Jersey (Table 2) Tajima’s *D* (*D*_*T*_) and Fu & Li’s *D* and *F* test statistics (*D*_*FL*_ and *F*_*FL*_), were significantly negative when calculated for all L4 isolates as a group (*n* = 47) and for the subgroup of non-S75 isolates from New York City. Negative values for *D*_*T*_ *, D*_*FL*_, and *F*_*FL*_ indicate a relative excess of low frequency alleles in a population, which can occur following recent population expansion or a selective sweep [52]. Fay and Wu’s *H*, a statistic that is insensitive to population expansion but highly sensitive to selection pressure, was not significantly different from zero for all isolate subgroups, suggesting that population expansion–rather than a selective sweep–explains the relative excess of rare alleles in isolates in L1-3 and non-S75 L4 isolates. Significant values for Ramos-Onsins & Rozas’ *R*_*2*_ statistic, which tests for recent population size expansion based on the difference between the number of singleton mutations and the mean number of nucleotide differences between samples, were observed for all subgroups except S75. All five neutrality test statistics were non-significant for the S75 outbreak cluster. Unlike other subgroups, the unfolded site frequency spectrum for S75 exhibited a lower number of low-frequency alleles (Fig. 3) and negative values for Fay and Wu’s *H* and normalized *H*, consistent with a small but non-significant excess of high-frequency derived alleles in this subpopulation.

### Purifying selection on genes involved in lipid metabolism and cell wall maintenance

dN/dS was significantly less than 1, consistent with purifying selection, for two genes in lipid metabolism pathways and five putative transmembrane protein genes (Supplementary Table S1). Two lipid metabolism pathway components, phenolphthiocerol synthesis polyketide synthase A–E family (*ppsA*) and polyketide synthase 12 (*pks12*), exhibited significantly decreased dN/dS in a specific subpopulation of New York City isolates (at nodes *b, c,* and *d* in Fig. 2). Evidence of episodic diversifying selection, with dN/dS significantly greater than 1, was limited to three isolates in our study, the L2 isolate W148, the L1 isolate T17, and the *M. africanum* K85 isolate.

## Discussion

*M. tuberculosis* exhibits very low sequence diversity compared to other bacteria, minimal evidence of horizontal gene transfer [53–55], and recombination limited to known highly variable gene families [28]. This lack of genetic diversity is pronounced in geographically restricted *M. tuberculosis* populations, such that locally endemic clone groups have posed a unique challenge to laboratory-based identification of TB outbreak clusters in New York City. Historically, these isolates have been strongly associated with homeless and at-risk populations, in which field epidemiology and contact tracing are often difficult, placing a premium on rapid and reliable laboratory identification of clustered cases. In one case series, 52% of patients infected with C strain isolates in NYC had no phone number or address, or could otherwise not be contacted by public health investigators [17]. The present study demonstrates how whole genome-based laboratory analysis can overcome these challenges, and suggests that WGS may be a particularly important tool at the local level, where genetic diversity is expected to be lower compared to more geographically diverse samples. The results presented here provide three specific approaches for identifying outbreak clusters and emerging strain groups in local *M. tuberculosis* populations: (1) genome-based phylogenetic reconstruction; (2) population genetic analysis, specifically estimation of neutrality and diversity statistics within grouped samples; and (3) genome-wide analysis for distinguishing signatures of purifying or diversifying selection.

Whole genome-based phylogenetic reconstruction yielded clearly defined population substructure among locally-endemic isolates in the New York City area, and identified S75 isolates as distinct clade in the phylogeny. S75 isolates also exhibited poorly differentiated terminal branching patterns, and relatively lower bootstrap support at individual nodes, which may reflect the limits of phylogenetic resolution inherent to available genome sequencing data. Although this approach allows for robust retrospective identification of outbreak clusters and emerging strain groups, it is perhaps less well suited for rapid identification of clustered transmission among new TB cases, in which low levels of genetic differentiation may preclude high-confidence phylogenomic resolution between isolates. However, as WGS-based technologies replace conventional genotyping methods, phylogenetic reconstruction will likely become an important tool in public health microbiology, providing a “phylogenetic reference” for TB isolates sequenced within a given geographic area or TB control program [56]. In addition, clustering of incident isolates in a specific phylogenetic branch could suggest ongoing transmission within a specific at-risk population.

SNP distance-based inference of recent transmission, in which the pairwise SNP distance is used to infer whether two isolates were transmitted directly between their respective hosts, is likely to become an important epidemiological tool in TB control [7, 57]. Although the distribution of pairwise SNP differences is expected to vary between low- and high-transmission areas (with higher average pairwise SNP differences expected in high-transmission settings and in areas with lower TB case notification rates) [58], emerging *M. tuberculosis* subpopulations are still expected to exhibit relatively few SNP differences between isolates. Identification of subpopulation-defining synapomorphic polymorphisms can support this approach by identifying unique SNPs shared between isolates in emerging subpopulations.

The two additional methods used in this study (estimation of neutrality and diversity statistics and selection analysis) are likely to have more value in retrospective analyses, where they can yield useful information about the epidemiological and evolutionary history of circulating *M. tuberculosis* subpopulations. Subgroup-defining polymorphisms can provide useful genetic markers for *M. tuberculosis* strain identification, similar to other minimal SNP sets used in *M. tuberculosis* phylogenetics [59]. S75 isolates in this study could be distinguished from other North American isolates using only 16 SNPs, and determination of similar subgroup-defining SNP sets could provide a straightforward tool for rapidly determining if a given TB isolate belongs to an existing outbreak cluster or endemic strain group. More broadly, subgroup-defining polymorphisms also provide interesting, if limited, insight into the evolutionary history of Lineage 4 *M. tuberculosis* isolates in North America and the specific L4 populations endemic to New York City. Isolates in these populations have only a minimal number of drug resistance-associated mutations, and instead have acquired multiple non-synonymous SNPs in virulence- and growth-associated pathways. Mutations in *pks5* and *yrbE2B* are of particular interest, first because of their well-characterized roles in *M. tuberculosis* virulence and second because they may both influence TNF-mediated host immune responses [39, 44]. S75 isolates strains are known to induce higher levels of TNF-α in vitro [60], which may help explain why S75 isolates have spread preferentially in immunocomproised patients. Although the functional consequences of these mutations are still unknown, these findings suggests that overall pathoadaptive fitness, rather than the acquisition of drug resistance mutations, may have played an important role in the evolutionary history of L4 *M. tuberculosis* populations in New York City.

Selection analysis identified two loci in *M. tuberculosis* lipid metabolism pathways, *ppsA* and *pks12*, with significantly decreased dN/dS ratios consistent with evolution under strong purifying selection. Observing signatures of purifying selection localized to a single subpopulation (in this case, the *M. tuberculosis* isolates grouped under Nodes *b, c,* and *d*), may suggest adaptation to a particular subpopulation or transmission niche, and thus provide useful information about risk factors for acquisition of infection with an emerging strain group. ppsA and other pps family genes are involved in the synthesis of phthiocerol and phenolphthiocerol, two components of cell wall lipids unique to pathogenic mycobacteria that likely participate in host-pathogen interactions [61] and virulence [62, 63]. Interestingly, Farhat et al. identified *ppsA* and *pks12* among 39 genes that exhibit signatures of convergence and possible positive selection in multidrug-resistant *M. tuberculosis* isolates [64]. Although these loci may exhibit signatures of positive selection in drug-resistant populations, it is not unexpected that *ppsA* and *pks12* would exhibit signatures of purifying selection in populations without a similar history of drug selection pressure. Consistent with this hypothesis we observed relatively fewer drug resistance-associated mutations in the same subpopulations where *ppsA* and *pks12* exhibit signatures of purifying selection. Furthermore we found that dN/dS ratios at known drug-resistance loci were not significantly greater than one in our sample, consistent with prior studies in drug-susceptible *M. tuberculosis* isolates [65]. The dN/dS ratio has limited power to detect positive selection in recently diverged intraspecific sequences and may underestimate the magnitude of negative selection in genes under strong purifying selection [66]. However, because the dN/dS ratio is expected to underestimate the magnitude of the selection coefficient in this context, our analysis is likely conservative, and the true magnitude of negative selection on *ppsA* and *pks12* may be larger than we have reported.

Lastly, estimation of multiple neutrality statistics yielded evidence for past population expansion across multiple subpopulations, consistent with prior studies on demographic expansion in *M. tuberculosis* populations [10, 67], with the notable exception of S75. This finding, in conjunction with the negative but nonsignificant *H* values estimated for S75 isolates (indicating an excess of high-frequency derived alleles), is consistent with the epidemiological history of this recently diverged group of closely related isolates. However, it is important to acknowledge that factors such as sample size and time since demographic expansion can influence the power of statistics that draw from the site frequency spectrum to detect past population growth. Specifically, site frequency spectrum-based statistics may fail to detect population expansion if the elapsed time since an expansion is either too small or too large, or with small sample sizes [52], and thus may be less useful for identification of emerging strain groups, as illustrated here. Importantly, the retrospective sample used in this study includes less than 0.01% of all *M. tuberculosis* infections occurring in New York City between 1999 and 2009 [2]. Nevertheless, this study demonstrates that even a small sample of isolates can yield meaningful information about the epidemiological and evolutionary history of endemic *M. tuberculosis* isolate groups in low-transmission settings.

## Conclusions

WGS-based technologies are likely to replace many conventional genotyping methods currently used in public health microbiology and TB epidemiology. How to maximize the public health value of this paradigm shift, and the large quantities of genomic data it will soon make available, is still an open question. Whole genome-based drug resistance profiling, SNP distance-based methods to identify ongoing transmission, and phylogenetic reconstruction will likely yield the most direct, practical benefits, and the WGS data collected during these activities will provide an important resource for ongoing research in TB epidemiology and pathogen evolution.

## Additional files

Additional file 1: Table S1. Complete list of non-synonymous and synonymous synapomorphic polymorphisms for all L4 isolates, L4 isolates from New York City and New Jersey, and isolates from the S75 outbreak cluster. (DOCX 19 kb)
Additional file 2:
**Figure S1.** Whole-genome maximum likelihood phylogenetic reconstruction of *Mycobacterium tuberculosis* isolates from North America, Sub-Saharan Africa, and Asia (*n* = 71). Values at the nodes indicate branch support based on 1000 bootstrap replicates. Letter labels denote branches with genes under purifying selection (see Additional file 1: Table S1) or lineage-defining polymorphisms. Green boxes in the adjoining matrix indicate SNPs at drug resistance-associated codon sites in known drug resistance gene. INH: isonaizid; RIF: rifampin; PZA: pyrazinamide; ETH: ethionamide; EMB: ethambutol; AMI: amikacin; FLQ: fluoroquinolones; PAS: para-aminosalicylic acid; SM: streptomycin. (PDF 210 kb)
Additional file 3:
**Table S2.** Statistically significant dN/dS values, estimated by gene and across phylogenetic branches. *P*_HOLM_, Holm-corrected *P*-value. Location of Nodes A–E are indicated in Fig. 1. (DOCX 19 kb)

## Abbreviations

MIRU: Mycobacterial interspersed repetitive units
SNP: Single nucleotide polymorphism
TB: Tuberculosis
WGS: Whole genome sequencing
