# Supplementary material for: Genomic epidemiology of Lineage 4 Mycobacterium tuberculosis subpopulations in New York city and New Jersey, 1999–2009
Source: BMC Genomics. 2016 Nov 21;17:947. doi: 10.1186/s12864-016-3298-6 (PMC5117616; doi:10.1186/s12864-016-3298-6)
Supplement: Additional file 1: Table S1. — Complete list of non-synonymous and synonymous synapomorphic polymorphisms for all L4 isolates, L4 isolates from New York City and New Jersey, and isolates from the S75 outbreak cluster. (DOCX 19 kb) [file 12864_2016_3298_MOESM1_ESM.docx]

| EPISODIC DIVERSIFYING SELECTION | | | | | | |  |  |
| --- | --- | --- | --- | --- | --- | --- | --- | --- |
| Gene | | **Branch** | **Lineage** | | **dN/dS** | **p_HOLM_** | **Class** | **Description** |
| Rv3236c | | W148 | 2 | | 1.25 | 0.00019 | Cell Wall and Cell Processes | Probable conserved membrane transport protein |
| Rv3616c | | T17 | 1 | | 6.05 | 0.01782 | Cell Wall and Cell Processes | Conserved hypothetical protein |
| Rv3433c | | W148 | 2 | | 1.32 | 0.00003 | Function Unknown | Conserved hypothetical protein |
| Rv2825c | | K85 | 6 | | 1.62 | 0.01753 | Function Unknown | Putative uncharacterized protein |
| Rv0058 | | K85 | 6 | | 1.48 | 0.00245 | Information Pathways | Replicative DNA helicase |
| Rv3580c | | W148 | 2 | | 1.37 | 0.00006 | Information Pathways: Translation | Cysteinyl-tRNA synthetase 1 *CysS1* |
| Rv3858c | | W148 | 2 | | 5.62 | 0 | Intermediary Metabolism/Respiration | Probable NADH-dependent glutamate synthase |
| Rv2196 | | K85 | 6 | | 1.24 | 0.00006 | Intermediary Metabolism/Respiration | Ubiquinol-cytochrome c reductase |
| Rv0101 | | K85 | 6 | | 2.51 | 0 | Lipid Metabolism | Probable peptide synthetase *nrp* |
| Rv2299c | | W148 | 2 | | 1.88 | 0.00005 | Molecular Chaperone | Probable chaperone protein *HtpG* |
| PURIFYING SELECTION | | | | | |  |  |  |
| Gene | **Branch** | | | **Lineage** | **dN/dS** | **p_HOLM_** | **Class** | **Description** |
| Rv0364 | W148 | | | 2 | 0.3 | 0.0002 | Cell Wall and Cell Processes | Possible conserved transmembrane protein |
| Rv1226c | W148 | | | 2 | 0.24 | 0.02824 | Cell Wall and Cell Processes | Possible conserved transmembrane protein |
| Rv3663c | W148 | | | 2 | 0.54 | 0 | Cell Wall and Cell Processes | Probable dipeptide ABC transporter *DPPD* |
| Rv2543 | W148 | | | 2 | 0.7 | 0 | Cell Wall and Cell Processes | Putative lipoprotein *lppA* |
| Rv2544 | W148 | | | 2 | 0.7 | 0.00011 | Cell Wall and Cell Processes | Putative lipoprotein *lppB* |
| Rv1157c | CDC1551 | | | 4 | 0.8 | 0.04155 | Function Unknown | Conserved hypothetical |
| Rv0650 | W148 | | | 2 | 0.32 | 0.00098 | Intermediary Metabolism/Respiration | Possible sugar kinase |
| Rv2931 | Node b | | | 4 | 0.19 | 0.00002 | Lipid Metabolism | Phenolpthiocerol synthesis polyketide synthase *ppsA* |
| Rv2931 | Node e­ | | | 4 | 0.31 | 0.00003 | Lipid Metabolism | Phenolpthiocerol synthesis polyketide synthase *ppsA* |
| Rv2931 | W148 | | | 2 | 0.29 | 0.00035 | Lipid Metabolism | Phenolpthiocerol synthesis polyketide synthase *ppsA* |
| Rv2048c | F11 | | | 4 | 0.77 | 0.00014 | Lipid Metabolism | Probable polyketide synthase *pks12* |
| Rv2048c | CDC1551 | | | 4 | 0.2 | 0 | Lipid Metabolism | Probable polyketide synthase *pks12* |
| Rv2048c | Node c | | | 4 | 0.24 | 0 | Lipid Metabolism | Probable polyketide synthase *pks12* |
| Rv2048c | Node d | | | 4 | 0.77 | 0.00014 | Lipid Metabolism | Probable polyketide synthase *pks12* |

**Additional file 1: Table S1.** Statistically significant dN/dS values, estimated by gene and across phylogenetic branches. *P*_HOLM_, Holm-corrected *P*-value. Location of Nodes A-E are indicated in Figure 1
