# Supplementary figures and images for: Genomic epidemiology of Lineage 4 Mycobacterium tuberculosis subpopulations in New York city and New Jersey, 1999–2009
Source: BMC Genomics. 2016 Nov 21;17:947. doi: 10.1186/s12864-016-3298-6 (PMC5117616; doi:10.1186/s12864-016-3298-6)

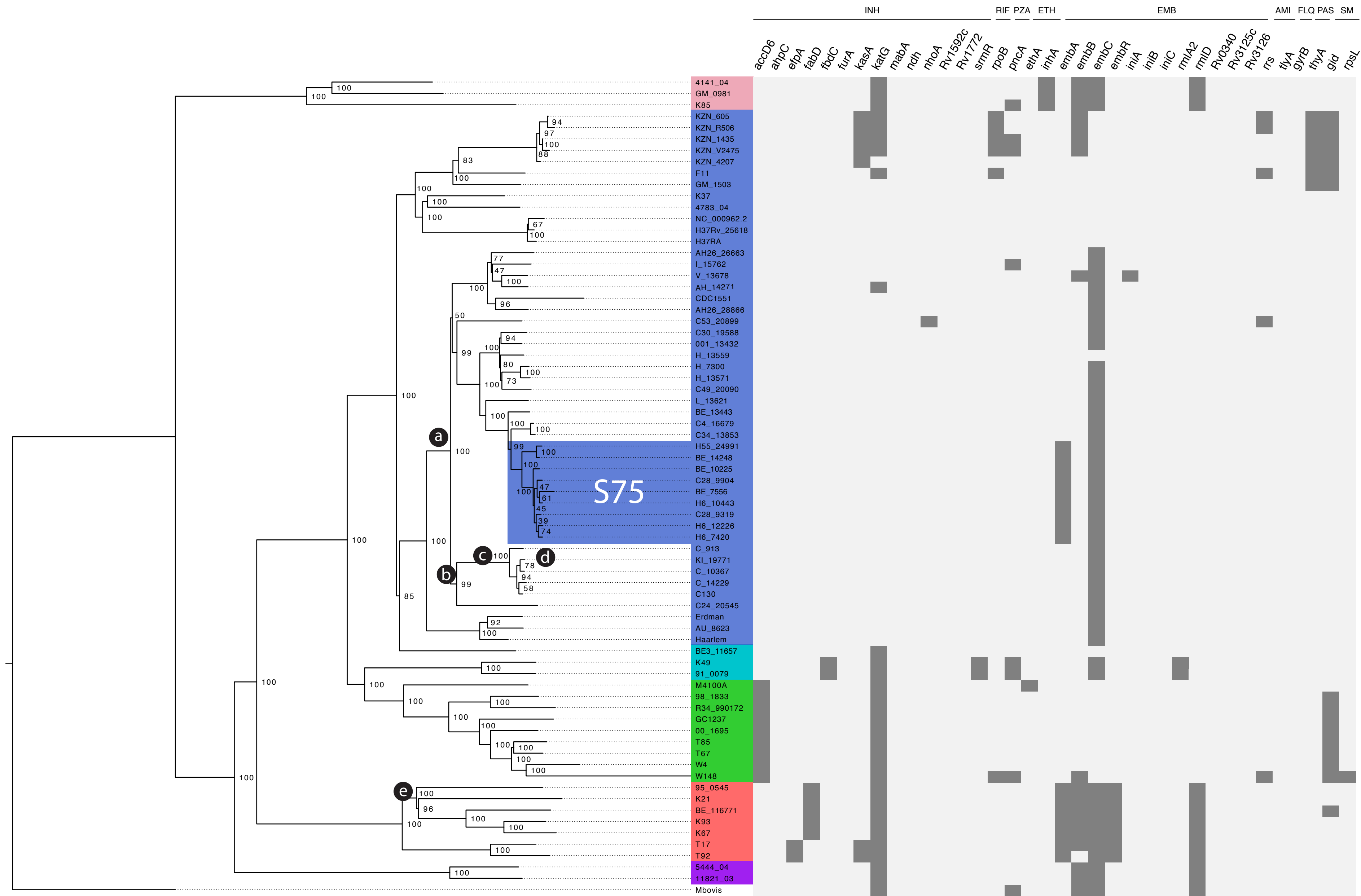

Supplement: Additional file 2: — Figure S1. Whole-genome maximum likelihood phylogenetic reconstruction of Mycobacterium tuberculosis isolates from North America, Sub-Saharan Africa, and Asia (n = 71). Values at the nodes indicate branch support based on 1000 bootstrap replicates. Letter labels denote branches with genes under purifying selection (see Additional file 1: Table S1) or lineage-defining polymorphisms. Green boxes in the adjoining matrix indicate SNPs at drug resistance-associated codon sites in known drug resistance gene. INH: isonaizid; RIF: rifampin; PZA: pyrazinamide; ETH: ethionamide; EMB: ethambutol; AMI: amikacin; FLQ: fluoroquinolones; PAS: para-aminosalicylic acid; SM: streptomycin. (PDF 210 kb) [file 12864_2016_3298_MOESM2_ESM.pdf]
